# A naturally light dilaton and a small cosmological constant

**DOI:** 10.1140/epjc/s10052-014-2790-x

**Published:** 2014-03-15

**Authors:** Brando Bellazzini, Csaba Csáki, Jay Hubisz, Javi Serra, John Terning

**Affiliations:** 1 Institut de Physique Théorique, CEA-Saclay, 91191 Gif-sur-Yvette Cedex, France; 2 Dipartimento di Fisica e Astronomia, Università di Padova and INFN, Sezione di Padova, Via Marzolo 8, 35131 Padua, Italy; 3 Department of Physics, LEPP, Cornell University, Ithaca, NY 14853 USA; 4 Department of Physics, Syracuse University, Syracuse, NY 13244 USA; 5 Department of Physics, University of California, Davis, CA 95616 USA

## Abstract

We present a non-supersymmetric theory with a naturally light dilaton. It is based on a 5D holographic description of a conformal theory perturbed by a close-to-marginal operator of dimension $4-\epsilon$ which develops a condensate. As long as the dimension of the perturbing operator remains very close to marginal (even for large couplings) a stable minimum at hierarchically small scales is achieved, where the dilaton mass squared is suppressed by $\epsilon$. At the same time the cosmological constant in this sector is also suppressed by $\epsilon$, and thus it is parametrically smaller than in a broken SUSY theory. As a byproduct we also present an exact solution to the scalar-gravity system that can be interpreted as a new holographic realization of spontaneously broken conformal symmetry. Even though this metric deviates substantially from AdS space in the deep IR it still describes a non-linearly realized exactly conformal theory. We also display the effective potential for the dilaton for arbitrary holographic backgrounds.

## Introduction

Dynamical spontaneous breaking of scale invariance (SBSI) is rare. If a theory is exactly conformal, it either does not break scale invariance, or the breaking scale is arbitrary (a flat direction) [1]. Thus an explicit breaking has to be present to trigger and stabilize the SBSI. However, this explicit breaking must remain small throughout the whole renormalization group running not to ruin scale invariance. In particular, the $\beta$-function of the coupling that introduces the explicit breaking must remain small at the scale of SBSI. This condition is difficult to satisfy: for example in QCD or technicolor (TC) models the condensates are triggered by large and rapidly changing couplings at the condensation scale $\Lambda _\mathrm{QCD,TC}$, implying large explicit breaking. Thus no light dilaton is expected in either case (in agreement with the absence of a light dilaton-like scalar hadron in QCD) [2, 3].

One possible scenario is that conformality is spontaneously broken along a flat direction, which is then lifted via the potential generated through a small external coupling whose non-zero but also small $\beta$-function breaks scale invariance explicitly. This mechanism is essentially what is assumed to happen in the Randall–Sundrum (RS) model [4] stabilized via the Goldberger–Wise (GW) mechanism [5]: the bulk scalar field is associated with the small and slowly running external coupling, and the appearance of the IR brane signals that SBSI has occurred [6, 7]. The resulting massive radion mode [8–11] is identified with the light dilaton of SBSI. However, in the 5D picture this scenario assumes that the IR brane tension has been tuned such that the radion potential is flat in the absence of the external perturbation. Generically such fine tunings are not present, and one would like to understand whether a light dilaton can still occur in the absence of tuning. In the 4D language the theory with a large mistune can be understood in terms of the dilaton potential in the following way [12–14]. Scale invariance allows an unsuppressed quartic (non-derivative) self-interaction term for the dilaton, since a dimension 4 operator in the action is scale invariant. An $O(1)$ mistune on the IR brane corresponds to a large quartic dilaton potential, which would generically prevent SBSI, at least for small perturbations. In the 5D picture a non-vanishing quartic would force the IR brane to infinity (and thus no SBSI), or the branes would be very close to each other (so effectively no scale invariant regime).

Contino, Pomarol and Rattazzi (CPR) have suggested in an important unpublished work [15] that this might be overcome if the quartic becomes mildly energy dependent via an explicit scale-invariance breaking perturbation, whose $\beta$-function remains parametrically small, but not necessarily the coupling itself. In this case the expectation is that SBSI will happen around the scale where the effective dilaton quartic vanishes, which can be a hierarchically small scale if the running lasts for a long time. At the same time the dilaton can be light, if the $\beta$-function is parametrically small at the scale of spontaneous breaking. The latter is the crucial dynamical assumption: the perturbation, which might start small in the UV, becomes sufficiently large in the IR to neutralize the initial large quartic, but at the same time its $\beta$-function must remain small. Phrased in a different way, as long as the $\beta$-function of the perturbation remains small, SBSI will naturally happen regardless of the absence of a flat direction to start with.

The mechanism of scanning through the possible values of the dilaton quartic coupling until it reaches the minimum where it almost vanishes is also important when we couple the theory to gravity. The value of the potential at the minimum corresponds to the cosmological constant contribution generated during the phase transition from a scale invariant theory to the broken phase. If the value of the potential at the minimum is naturally suppressed by the smallness of the $\beta$-function in the IR, the contribution to the cosmological constant could be significantly reduced. This is a mechanism along the lines Weinberg was considering in [16], except that he was requiring that the cosmological constant vanishes exactly, which in turn requires an exactly vanishing $\beta$-function. However, in this case no dilaton stabilization can happen. The potential significance of the dilaton for reducing the cosmological constant was also emphasized in [17].

The aim of this paper is to examine the CPR proposal in a holographic setting and establish that it can indeed be a viable route towards finding a parametrically light dilaton in a dynamical SBSI theory with hierarchical scales. We will argue that even though the metric can deviate significantly from AdS space, this is due to the formation of a condensate of the perturbing operator which is very close to dimension 4. As long as the dimension is very close to marginal ($4-\epsilon$), the condensate will correspond to pure spontaneous breaking of scale invariance, with the resulting contribution to the dilaton potential still corresponding to a quartic (which will, however, acquire a mild scale dependence due to the running, $\epsilon \ne 0$). Moreover, as long as the running is slow, $\epsilon \ll 1$, the condensation in the IR will be universal: it will not depend on the details of the exact form of the $\beta$-function (which is captured by the form of the bulk scalar potential). Therefore in the IR the solutions to the coupled tensor–scalar equations will well be approximated by the exact solution to the system with a dimension 4 condensate (corresponding to the case of no bulk scalar potential aside from the negative cosmological constant). In the UV a slowly running solution perturbing the AdS background can be used. These solutions can be joined using asymptotic matching.^1^ This way we will be able to explicitly calculate the effective dilaton potential and show that the mass is suppressed by the small parameter $\epsilon$ of the $\beta$-function at the minimum of the potential. This yields an explicit construction for a dilaton that is parametrically lighter than the dynamical scale of the theory as required for models where the dilaton is a Higgs-like particle [12–14, 19–21]. Moreover, we show that the value of the dilaton potential at the minimum, which provides the cosmological constant contribution from the phase transition, is also suppressed by $\epsilon$, which for realistic hierarchies can be as small as $\epsilon \sim 10^{-2}$. On the way we present an exact solution to the scalar-gravity system which is the gravity dual of a dimension 4 operator condensing in the IR, thereby yielding a fully spontaneous breaking of scale invariance. Even though the scalar background is not flat, and the deviation of the metric from AdS is large in the IR, this theory still realizes an exactly conformal theory that is spontaneously broken.

CPR also comment on the nature of the bulk scalar and the origin of its suppressed potential: a large coupling with a small $\beta$-function in 4D may be the dual of a 5D Goldstone boson of the bulk with the potential suppressed by the Goldstone shift symmetry. Of course, other realizations of small $\beta$-functions can be envisioned as well, e.g. the coupling approaching a strongly interacting IR fixed point that is not reached because of *early* condensation. The construction presented here can be thought of as the proper realization of walking in technicolor theories [22–26]: in order to obtain a light dilaton the $\beta$-function needs to remain small even at the scale where the condensates are generated. In the following we will not actually need to commit to any specific realization and the only crucial assumption is that the bulk potential is suppressed by a small symmetry breaking parameter.

The paper is organized as follows: in Sect. 2 we give an overview of the mechanism for obtaining a light dilaton and in particular emphasize the differences between the standard GW picture and the CPR proposal. In Sect. 3 we show how to calculate the dilaton effective potential in general holographic theories where the metric could deviate from AdS significantly. Section 4 is devoted to the discussion of the solution with a dimension 4 condensate (vanishing bulk scalar mass), and of how to obtain a flat dilaton potential in that case via tuning two condensates against each other. Finally in Sect. 5 we show how a naturally light dilaton can be obtained via the introduction of the small bulk mass, and we comment on the suppression of the resulting cosmological constant in that case. Several appendices are devoted to an alternative derivation of the dilaton effective potential (Appendix A), the detailed derivation of the small back-reaction case (Appendix B) and the GW case (Appendix C), an explanation of the asymptotic matching procedure for the boundary layer problem used for finding the full solution (Appendix D), a discussion of the dilaton kinetic term as well as dilaton parametrizations (Appendix E), and finally a discussion of an alternative choice for the IR brane potential (Appendix F).

## Light dilatons via long running and small $\beta$-function

Unlike for internal symmetries, non-linearly realized spontaneously broken scale invariance allows a non-derivative quartic self-interaction for the dilaton:

2.1 $$\begin{aligned} V_\mathrm{eff} = F \chi ^4 \end{aligned}$$

where $\chi$ is the dilaton field with scaling dimension 1. For a theory without explicit breaking one needs to have $F=0$ in order for SBSI to occur: if $F>0$, the minimum is at $\chi =0$ (no SBSI), while for $F<0$ we find $\chi \rightarrow \infty$, thus there is no scale invariant theory. So the only possibility is that $F=0$, and thus $\chi$ is a flat direction: just like the flat potential valley for ordinary Goldstone bosons, the main difference being that the dilaton corresponds to a non-compact flat direction. If one wants to stabilize the scale one needs to introduce a small explicit breaking by perturbing the theory with a close-to-marginal operator $\mathcal{O}$ with a slowly running coupling $\lambda$. This will generate a small non-trivial potential

2.2 $$\begin{aligned} V_\mathrm{eff} = \chi ^4 F(\lambda (\chi )), \quad F(\lambda =0) \sim 0, \end{aligned}$$

which can introduce a non-trivial minimum for the potential at hierarchically small dilaton values, and which will give rise to a small dilaton mass.

$F=0$ and the appearance of a flat direction is natural in supersymmetric theories. Focusing on non-supersymmetric theories, one may ask how likely it is for $F\sim 0$ to occur in any given theory. The simplest answer is to perform an NDA analysis in the low-energy effective theory for the dilaton which gives an estimate for the size of the quartic [12] $F\sim 16 \pi ^2$. From this point of view spontaneous scale symmetry breaking looks quite unlikely and can be tuned at best in non-SUSY theories. This issue is even more evident if we notice that by reparametrizing the dilaton as $\chi =f \hbox {e}^{\sigma /f}$ with $\langle {\sigma } \rangle =0$, the question of $F=0$ is reminiscent of a vanishing cosmological constant, $\Lambda _\mathrm{eff} = F f^4$.

**Fig. 1:**
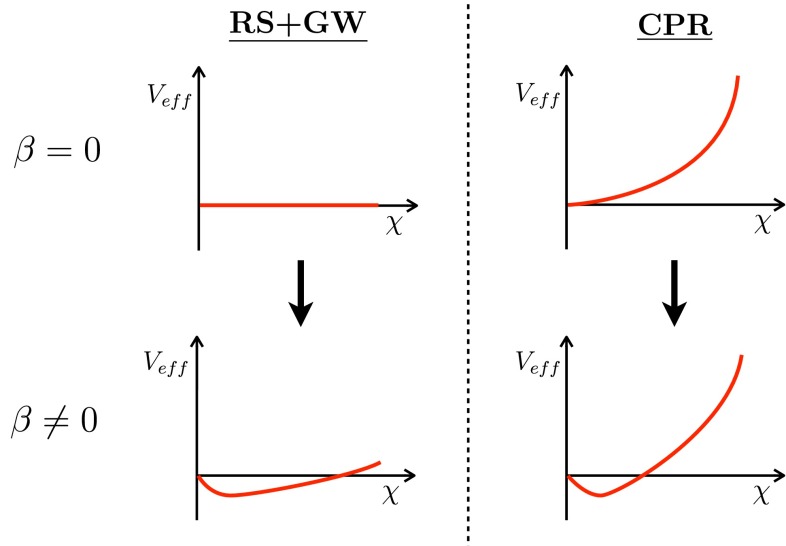
Pictorial representation of the tuned scenario with vanishing quartic in the absence of stabilizing perturbation (*left*) versus the proposal discussed in this work, where a large perturbation compensates for the large initial quartic (*right*)

Contino et al. [15] have, however, suggested a different viewpoint: the presence of a flat direction (in the absence of perturbation) is not required (nor is it natural). Their approach is then that a theory with $F\ne 0$ will simply not break scale invariance spontaneously. Thus for a successful breaking of scale invariance a theory needs to be able to scan its value of $F$, until $F\sim 0$ is reached. In effect one needs a scale dependent quartic $F(\mu )$, which can be achieved by introducing an external coupling $\lambda$, explicitly breaking scale invariance via its running,

2.3 $$\begin{aligned} \frac{\hbox {d}\lambda }{\hbox {d}\log \mu } = \beta (\mu ) \equiv \epsilon \, b(\lambda ) \ll 1, \end{aligned}$$

where $b(\lambda )$ is a generic function of $\lambda$, whose detailed form is not important as long as the small parameter $\epsilon$ can be factored out. This running coupling will in effect adjust the value of $F$ from its UV value (presumably of order $\sim$ $16 \pi ^2$). If sufficiently long running is allowed, the corrections $\delta F \sim (\Lambda _\mathrm{UV}/\mu )^\epsilon$ can become sizable, and at some scale $\mu _\mathrm{IR}$ we find $F(\lambda (\mu _\mathrm{IR}) ) \sim 0$. At this scale spontaneous breaking of scale invariance can happen. Since scale invariance is effectively recovered by substituting $\mu \rightarrow \chi$, this mechanism is equivalent to the generation of a non-trivial potential for the dilaton, Eq. (2.2), but with $F(\lambda = 0 ) \sim 16 \pi ^2$, and with its minimum determined by $F(\lambda (\chi )) \sim 0$. Thus the CPR idea is to let the theory scan through the values of $F$ driven by the small explicit breaking term. The running will stop when the critical value $F \sim 0$ is reached and spontaneous breaking of scale invariance will occur. The differences between the scenario with $F \sim 0$, to which we refer as RS$+$GW (recalling its extra-dimensional realization), and $F \sim 16 \pi ^2$ are illustrated in Fig. 1. It is of course very important that the explicit breaking of scale invariance, that is, the $\beta$-function, remains very small all throughout the running, and in particular at the IR scale where $F \sim 0$, otherwise the dilaton would pick up a large mass. This is exactly what happens in QCD or in technicolor: one starts out with a small $\beta$-function and an approximately conformal theory in the UV. However, in the IR the coupling and $\beta$ become large, and thus at energies where the QCD (or techniquark) condensates form there is no longer an approximate scale invariance and hence no light dilaton is expected, in accordance with the absence of an additional light scalar in QCD.

In order for the scanning mechanism to be possible, the contribution of the perturbation must approximately cancel the existing large tree-level quartic in the dilaton potential. This can happen only if the value of the coupling of the perturbing operator eventually becomes large. That does not automatically imply a large dilaton mass as long as the $\beta$-function remains small even while the coupling, $\lambda$, itself is big. This cancelation can be understood as follows: the increase in $\lambda$ along the running will be accompanied by a condensate for the perturbing operator $\mathcal{O}$, which will contribute a term $\propto \chi ^{4-\epsilon }$ to the dilaton potential. If $\epsilon$ is very small (that is, the condensate $\langle \mathcal{O}\rangle$ is very close to dimension 4) this term can cancel the existing tree-level quartic at a hierarchically smaller scale than where the running started, and it can allow the CFT operators to also condense, giving rise to SBSI. Thus in this case the external perturbation both triggers and stabilizes SBSI. One can see that for this to work it is essential for $\mathcal{O}$ to be very close to dimension 4, that is, $\epsilon \ll 1$ throughout the running and even when $\lambda$ becomes sizable at the condensation scale.

The issue of whether a theory can scan through the possible values of $F$ and settle at a minimum where $F\sim 0$ is particularly interesting since in a gravitational theory the value of $F$ at the minimum corresponds to the cosmological constant generated during the phase transition from the scale invariant to the broken phase. If $F\sim 0$ is natural in a theory coupled to gravity then a large (and perhaps most problematic) part of the total cosmological constant could be significantly reduced. The full cosmological constant in a model with an approximately conformal sector giving rise to electroweak symmetry breaking is made up of

2.4 $$\begin{aligned} V_\mathrm{tot}=V_\mathrm{UV}+V_\mathrm{TeV}+V_\mathrm{IR} \end{aligned}$$

where $V_\mathrm{UV}$ is the value of the cosmological constant at the UV cutoff scale, $V_\mathrm{TeV}$ is the contribution of the broken conformal sector (generically expected to be of the size $(\hbox {TeV})^4$ and contains the contribution from electroweak symmetry breaking), while $V_\mathrm{IR}$ is the contribution due to all low-scale phase transitions well below the electroweak scale (for example due to the QCD phase transition). In a holographic model $V_\mathrm{UV}$ would correspond to the contribution of the UV degrees of freedom localized on or around the UV brane, $V_\mathrm{TeV}$ to that of the degrees of freedom localized on or around the IR brane, while $V_\mathrm{IR}$ is the contribution from physics below the scale of the lightest bulk KK mode or radion mode, where the theory effectively becomes 4 dimensional. One could perhaps imagine eliminating $V_\mathrm{UV}$ via high scale SUSY, with a non-trivial interplay between SUSY and the conformal symmetry (for example SUSY might only be broken on the IR brane). Another possibility would be to use the hierarchically small dilaton VEV only to solve a little hierarchy between 10 and 1 TeV, while above 10 TeV the theory becomes supersymmetric. In the model with dynamical SBSI triggered by the field of dimension $4-\epsilon$ discussed above, the expectation is that the value of the minimum of the potential will be suppressed by $\epsilon$ (since for $\epsilon \rightarrow 0$ the entire potential is again a pure quartic that forces $\chi = 0$), thus

2.5 $$\begin{aligned} V_\mathrm{TeV} \sim \epsilon (\hbox {TeV})^4. \end{aligned}$$

Finally, the contribution of the IR scale physics is expected to be of order $$V_\mathrm{IR} \sim m_\mathrm{dil}^4/(16 \pi ^2)$$, set by the size of the dilaton mass. If QCD was composite and the dilaton mass is smaller than the QCD scale then the energy from the QCD phase transition would be incorporated to a contribution to $$F$$ and already be part of the estimate in (2.5). If the dilaton mass is heavier than the QCD scale then there will already be loop contributions to the cosmological constant in the 4D theory above the QCD scale which will be the leading contributions to $$V_\mathrm{IR}$$. The dilaton mass (as we will see later) is expected to scale with $$\epsilon $$ as $$m_\mathrm{dil}^2 \propto \epsilon (\hbox {TeV})^2$$, thus the leading cosmological constant is given by (2.5). In order to reduce this to observed magnitudes one would need $$\epsilon \lesssim 10^{-60}$$. The associated approximately massless dilaton would mediate a long range force similar to gravity, with strength $$\propto 1/(\hbox {TeV})^2$$ [17]. Fifth force bounds require that $$\epsilon \gtrsim 10^{-24}$$ [27, 28] (corresponding to $$m_\mathrm{dil}\gtrsim 6$$ eV). Dilatons lighter than a few hundred MeV will decay to photons. The lifetimes of dilatons with mass above an MeV are shorter than a second and are not expected to influence early Universe cosmology. Dilatons lighter than an MeV could significantly affect BBN and a detailed study would be needed to decide whether a scenario with ultra-light dilatons remains viable. However, there is another important reason not to consider the case with ultra-light dilatons. As we will show in Sect. 5, there is a correlation between the Planck-weak hierarchy and the amount of running in this scenario: the Planck-weak hierarchy is given by the IR brane mistuning raised to the power $$1/\epsilon $$. For $${\mathcal {O}}(1)$$ mistunes, the observed hierarchy implies $$\epsilon \sim 1/\log (M_\mathrm{Pl}/M_\mathrm{weak}) \sim 0.1 $$–$$ 0.01$$: an $$\epsilon $$ significantly smaller than this would require more running (and hence a much bigger $$M_\mathrm{Pl}$$) to settle at the point with a small quartic. For a fixed hierarchy this can be understood as an issue of initial conditions. Given the hierarchy one needs to be a distance $$\Delta \lambda \sim \epsilon \log \frac{M_\mathrm{Pl}}{M_\mathrm{weak}}$$ from the solution of $$F(\lambda _0) \sim 0$$. For exponentially small $$\epsilon $$ (needed to significantly reduce the cosmological constant) one needs to start exponentially close to the tuned value of the coupling $$\lambda $$, while for $$\epsilon \sim 0.1$$–$$0.01$$ one can be $$\mathcal{O}(1)$$ away from the special point. The conservative option is then to assume $$\epsilon \sim 0.1$$–$$0.01$$ and that Eq. (2.5) is tuned against $$V_\mathrm{UV}$$ to yield the observed cosmological constant.

Weinberg has argued [16] that a dilaton-like field can not be used to relax the cosmological constant to zero: if the theory is exactly conformal ($\epsilon =0$) the dilaton does not get stabilized, and one needs tuning to set the cosmological constant to zero, while for a broken theory ($\epsilon \ne 0$) the cosmological constant is not zero. This is not in contradiction with the arguments here. We will indeed see that for $\epsilon = 0$ one needs to tune the parameters of the theory in order to obtain a flat dilaton (and a vanishing low-energy contribution to the cosmological constant). However, we will see that for $\epsilon \ne 0$ the theory can relax to a vacuum with a small ($\epsilon$-suppressed) vacuum energy.

## The dilaton effective potential in holographic models

A general holographic model can be obtained by considering the action

3.1 $$\begin{aligned} S&= \int \hbox {d}^5 x \sqrt{g} \left( - \frac{1}{2 \kappa ^2} \mathcal {R} + \frac{1}{2} g^{MN} \partial _M \phi \partial _N \phi - V(\phi ) \right) \nonumber \\&-\int \hbox {d}^4x \sqrt{g_0} V_0 (\phi ) - \int \hbox {d}^4x \sqrt{g_1} V_1 (\phi ) \end{aligned}$$

of a bulk scalar field $\phi$ coupled to gravity. Here $\kappa ^2$ is the 5D Newton constant, which is related to 5D Planck scale via $\kappa ^2 = \frac{1}{2 M_*^3}$. We will be considering 4D Lorentz invariant solutions to the Einstein equations, thus our metric ansatz will be

3.2 $$\begin{aligned} \hbox {d}s^2 = \hbox {e}^{-2 A(y)}\,\hbox {d}x^2 - \hbox {d}y^2, \end{aligned}$$

where $\hbox {e}^{-A(y)}$ is the general warp factor. The AdS/CFT prescription gives an identification between the extra-dimensional coordinate and an energy scale in a dual 4D CFT:

3.3 $$\begin{aligned} \mu =k \hbox {e}^{-A(y)}, \end{aligned}$$

where $k = \sqrt{ \frac{-\Lambda _{(5)}\kappa ^2}{6} }$ is the curvature of the AdS space, determined by the 5D cosmological constant $\Lambda _{(5)}$.

We can then calculate the effective potential for the dilaton for an arbitrary background. We will assume that the general background is cut off at the position $y=y_1$ with orbifold boundary conditions, which corresponds to the presumed spontaneous breaking of conformality. The dilaton is identified as the scale of the spontaneous breaking, which in this case corresponds to the IR brane position $y_1$, implying

3.4 $$\begin{aligned} \chi = \hbox {e}^\frac{\sigma }{f} = \hbox {e}^{-A(y_1)}. \end{aligned}$$

Both $\mu$ and $\chi$ are identified up to an unphysical arbitrary constant, $A(y) \rightarrow A(y) + a$ being a symmetry of the system. We will fix it by requiring $A(0)=0$. Besides, reparametrizations of the dilaton field should not change physical quantities, and when convenient we will simply take $\chi = \hbox {e}^{-ky_1}$ (see also Appendix E).

The background has to solve the bulk equations of motion

3.5 $$\begin{aligned} 4 A^{\prime 2}-A^{\prime \prime }&= -\frac{2\kappa ^2}{3} V(\phi ) \nonumber \\ A^{\prime 2}&= \frac{\kappa ^2 \phi ^{\prime 2}}{12}-\frac{\kappa ^2}{6} V(\phi ) \nonumber \\ \phi ''&= 4 A' \phi ' +\frac{\partial V}{\partial \phi }. \end{aligned}$$

The BCs (assumed to be $Z_2$-symmetric) are then

3.6 $$\begin{aligned} 2 A'|_{y=y_0,y_1}&= \pm \frac{\kappa ^2}{3} V_{0,1} (\phi )\Bigg |_{y=y_0,y_1,} \end{aligned}$$

3.7 $$\begin{aligned} 2\phi '|_{y=y_0,y_1}&= \pm \frac{\partial V_{0,1}}{\partial \phi }\Bigg |_{y=y_0,y_1,} \end{aligned}$$

where the $+$ sign is for the UV brane and the $-$ sign for the IR brane.

Let us now calculate the effective potential for the dilaton in these general backgrounds. The effective potential is obtained by integrating the bulk action over the solutions of the bulk equations of motion, with the scalar BCs (3.6) imposed both at the UV and the IR. We do not impose the Israel junction conditions (3.6) corresponding to the BC for the warp factor. Eventually the UV brane junction condition can be imposed, thereby fixing the location $y_0$ of the UV brane, and possibly at the price of tuning the UV brane tension. The effective potential in terms of the general warp factor $A(y)$ and the general scalar background $\phi (y)$ is then given by

3.8 $$\begin{aligned} V_\mathrm{eff}(\chi )&= -2 \int _{y_0}^{y_1} \hbox {d}y \sqrt{g} \left[ \!-\!\frac{1}{2\kappa ^2} (20 A^{\prime 2} \!-\!8 A'') \!-\!\frac{1}{2} \phi ^{\prime 2}\!-\!V(\phi )\right] \nonumber \\&+\sqrt{g} V|_0 +\sqrt{g} V|_1. \end{aligned}$$

Here we have integrated over the full circle rather than just over the orbifold. Special attention has to be paid to the singular pieces in $A^{\prime \prime }$ at the two boundaries, which will give an additional contribution to the effective potential of

3.9 $$\begin{aligned} V_\mathrm{eff}^\mathrm{(sing)} = \left[ \sqrt{g} \frac{8 A'}{\kappa ^2}\right] _0^1, \end{aligned}$$

while using the bulk equations of motion in (3.5) the smooth part of the bulk is given by

3.10 $$\begin{aligned} V_\mathrm{bulk}&= \frac{2}{\kappa ^2} \int _{y_0}^{y_1}\,\hbox {d}y\, \hbox {e}^{-4A(y)} (4 A^{\prime 2}-A'')\nonumber \\&= -\left[ \sqrt{g} \frac{2}{\kappa ^2} A' \right] _0^1. \end{aligned}$$

As expected, the entire effective potential is a boundary term, given in terms of the location of the IR brane $y_1$ by

3.11 $$\begin{aligned} V_\mathrm{eff}=V_\mathrm{UV}+V_\mathrm{IR}, \end{aligned}$$

with

3.12 $$\begin{aligned} V_\mathrm{UV/IR}\!=\! \hbox {e}^{-4 A(y_{0,1})} \left[ V_{0,1} \left( \phi (y_{0,1})\right) \mp \frac{6}{\kappa ^2} A' (y_{0,1} )\right] \!.\quad \end{aligned}$$

An alternative derivation of this effective potential using the Gibbons–Hawking boundary action is given in Appendix A. As expected, this potential vanishes for a solution that actually satisfies the boundary conditions (3.6) which we have not yet imposed. Once those are satisfied one has a flat solution to the bulk equations of motion and the resulting effective 4D cosmological constant necessarily vanishes. This does not mean that the entire potential identically vanishes, nor does it imply that the minimum of the potential has to be at zero. In terms of the dilaton field $\chi = \hbox {e}^{-A(y_1)}$ and the location of the UV brane $\mu _0 = \hbox {e}^{-A(y_0)}$ (which effectively acts as UV cutoff regulator), the effective potential is

3.13 $$\begin{aligned} V_\mathrm{IR}&= \chi ^4 \left[ \! V_1 \left( \phi \left( A^{-1} (\!-\!\log \!\chi )\right) \right) \!+\!\frac{6}{\kappa ^2} A'\left( \! A^{-1} (\!-\!\log \chi )\right) \right] ,\nonumber \\ \end{aligned}$$

while $V_\mathrm{UV}$ is obtained by $\chi \rightarrow \mu _0$ and a sign flip in front of the $A'$ term. The form of this potential is in accordance with the expectation that the general dilaton potential of a spontaneously broken conformal theory should be of the form [12]

3.14 $$\begin{aligned} V_\mathrm{eff}(\chi )= \chi ^4 F(\lambda (\chi )), \end{aligned}$$

where $\lambda$ is a coupling that introduces an explicit breaking of scale invariance. Therefore we can make the holographic identification

3.15 $$\begin{aligned} F = V_1 + \frac{6}{\kappa ^2} A'. \end{aligned}$$

In the case of pure spontaneous breaking the potential should just be a pure quartic, which must vanish if there is a stable vacuum in which scale invariance is spontaneously broken. For example in the case of pure AdS space without a scalar field (the original RS1 setup) the effective potential is indeed a pure quartic. In this case, we have $A'=k$, $V_1 (\phi ) =\Lambda _1$ (the IR brane potential is just a pure tension), and the effective dilaton potential is

3.16 $$\begin{aligned} V_\mathrm{dil, RS}= \chi ^4 \left( \Lambda _1+\frac{6k}{\kappa ^2} \right) . \end{aligned}$$

This pure quartic must vanish for the IR brane to not fly away or collide with the UV brane. From the 5D point of view the vanishing of this quartic is interpreted as the second fine tuning of RS.

The minimization condition of the dilaton potential Eq. (3.14) can be written as

3.17 $$\begin{aligned} \left. \frac{\hbox {d}V_\mathrm{eff} (\chi )}{\hbox {d}\chi } \right| _{\chi =\langle {\chi } \rangle } = 0, \end{aligned}$$

with

3.18 $$\begin{aligned} \frac{\hbox {d} V_\mathrm{eff} (\chi )}{\hbox {d} \chi } = \chi ^3\left[ 4 F + \frac{\partial F}{\partial \lambda } \beta \right] , \quad \beta = \frac{\partial \lambda }{\partial \log \chi }. \end{aligned}$$

Since we will require that the potential is minimized, we see that at the minimum

3.19 $$\begin{aligned} F = -\frac{1}{4} \frac{\partial F}{\partial \lambda } \beta , \end{aligned}$$

implying that the potential at the minimum will be proportional to the value of the $\beta$-function. We will derive explicitly the same result from Eq. (3.13) in Sect. 5. That the value at the minimum itself might be non-vanishing implies that the solution does not actually have flat 4D sections, therefore to find the corresponding complete bulk solution a more general ansatz, different from (3.2), would be needed, along the lines of [29].

## Constant bulk potential–flat dilaton potential by tuning two condensates

Before we discuss the case with a non-trivial scalar bulk potential, it is very instructive to consider the theory with a constant potential. This is useful for two reasons:

- It provides a 5D gravity dual for the formation of a dimension 4 condensate and hence a ‘soft-wall’ version of the RS-model of SBSI.
- This solution will be relevant for the IR region for the discussion of the general case with a small bulk mass in the next section.

The theory with constant bulk potential corresponds to adding an additional exactly marginal operator to the theory. If this operator condenses, it is expected to give another $\chi ^4$ quartic term to the dilaton potential. For the case with a finite UV brane one also generically expects additional terms suppressed by the UV scale $\mu _0$. This will provide us with an alternative way of obtaining a flat dilaton potential compared to RS/GW. In GW one tunes the IR brane tension against the bulk cosmological constant to ensure that the condensate corresponding to the IR brane does not produce a quartic dilaton term, resulting in a flat dilaton potential. The other possibility considered here is to not impose the RS tuning at the IR brane, allowing a tree-level quartic from the condensate, but then canceling this with another quartic corresponding to the condensate of the bulk scalar. By appropriately tuning the two condensates against each other one finds another way of obtaining a flat dilaton potential. While this also involves tuning, the significance of this is that by introducing the small bulk mass this tuning can be alleviated.

We parametrize the bulk potential as

4.1 $$\begin{aligned} V(\phi ) = \Lambda _{(5)} = -\frac{6k^2}{\kappa ^2}. \end{aligned}$$

For concreteness we will choose quadratic brane potentials,

4.2 $$\begin{aligned} V_i(\phi ) = \Lambda _i + \lambda _i (\phi - v_i)^2, \end{aligned}$$

though for most arguments the detailed form of the brane potentials will not matter. The bulk only depends on the derivative of the scalar field, and thus one has a $\phi \rightarrow \phi +C$ shift symmetry, which signals the presence of conformal symmetry in this case. Thus one expects this to correspond to a purely spontaneous breaking of scale invariance.

The bulk equations of motion for this case can be solved analytically and the solutions are [30]

4.3 $$\begin{aligned} A(y)&= -\frac{1}{4} \log \left[ \frac{\sinh 4k (y_\mathrm{c}-y)}{\sinh 4k y_\mathrm{c}} \right] ,\end{aligned}$$

4.4 $$\begin{aligned} \phi (y)&= -\frac{\sqrt{3}}{2\kappa } \log \tanh [2k (y_\mathrm{c}-y)] +\phi _0 . \end{aligned}$$

In this expression the (unphysical) constant in the warp factor was fixed such that $A(0)=0$. This solution describes the formation of a 4-dimensional condensate corresponding to the operator $\mathcal{O}$ that $\phi$ couples to. The singularity at $y_\mathrm{c}$ corresponds to this condensate. This solution on its own can be considered a ‘soft-wall’ version of a model of SBSI. While RS corresponds to the condensation of an infinite-dimensional operator (hence the metric is exactly AdS all the way till the condensate forms, described by the appearance of the IR brane), here we have the more realistic case of the formation of a dimension 4 condensate. Both of these correspond to pure spontaneous breaking of scale invariance, and hence both of these should give pure quartic potentials for the dilaton. In our construction we will assume that both condensates are present, and that the pure RS condensate forms earlier, hence the IR brane will shield the singularity. Therefore we consider the region $y<y_\mathrm{c}$, and the location of the IR brane $y_1$ appears before the singularity, $y_1<y_\mathrm{c}$: the RS condensate in the CFT forms at a higher energy scale than the $\mathcal{O}$ condensate.

For finite $y_\mathrm{c}$, the AdS boundary is at $y=- \infty$,

4.5 $$\begin{aligned} A'(y \rightarrow - \infty )=k , \quad \phi (y \rightarrow - \infty ) = \phi _0 . \end{aligned}$$

Exact AdS space is only obtained in the limit $y_\mathrm{c} \rightarrow \infty$,

4.6 $$\begin{aligned} \lim _{y_\mathrm{c} \rightarrow \infty } A'(y) = k , \quad \lim _{y_\mathrm{c} \rightarrow \infty } \phi (y) = \phi _0 . \end{aligned}$$

The scalar profile is constant in this limit. The AdS limit Eq. (4.6) can only be obtained by imposing the requirement that both brane potentials are pure tensions (no $\phi$-dependence) and the tensions obey the RS tunings:

4.7 $$\begin{aligned} V_i(\phi ) = \mp \frac{\Lambda _{(5)}}{k}, \end{aligned}$$

in which case the singularity is pushed to $y_\mathrm{c}\rightarrow \infty$.

For generic brane potentials $y_\mathrm{c}$ will be finite, thus the space will deviate from pure AdS. We want to find the effective potential for the dilaton field in this case. A convenient parameterization of the dilaton $\chi$ and the location of the UV brane $\mu _0$ is

4.8 $$\begin{aligned} \begin{aligned} \chi ^4&= \hbox {e}^{-4 A(y_1)} = \frac{\sinh 4k(y_\mathrm{c}-y_1)}{\sinh 4ky_\mathrm{c}}, \\ \mu _0^4&=\hbox {e}^{-4 A(y_0)}= \frac{\sinh 4k(y_\mathrm{c}-y_0)}{\sinh 4ky_\mathrm{c}} , \end{aligned} \end{aligned}$$

while for the location of the singularity we will use the parametrization

4.9 $$\begin{aligned} \delta ^4 = \frac{1}{\sinh 4ky_\mathrm{c}}. \end{aligned}$$

To determine the effective potential we need to impose the BCs for the scalar field Eq. (3.7). For concreteness we can choose simple quadratic brane potentials Eq. (4.2), though the specific form of the brane potentials will not be important. For these potentials the scalar boundary conditions are

4.10 $$\begin{aligned}&2 \lambda _i \left( \phi _0 -\frac{\sqrt{3}}{2\kappa } \log \tanh [2k(y_\mathrm{c}-y_i)] -v_i\right) \nonumber \\&\quad = \mp \frac{2\sqrt{3}k}{\kappa } \frac{1}{\sinh 4k(y_\mathrm{c}-y_i)}. \end{aligned}$$

These should be used to determine the constants $y_\mathrm{c}$ and $\phi _0$ for use in the effective potential. Since both of these equations depend only on the distances of the brane to the singularity $y_i-y_\mathrm{c}$ both of them can be written in terms of the combination of the variables $\chi ^4/\delta ^4$ and $\mu _0^4/\delta ^4$. We can use the UV scalar equation to determine $\phi _0$ in terms of the location of the UV brane as

4.11 $$\begin{aligned} \phi _0 = v_0 \left( 1+ f_0 (\delta ^4/\mu _0^4)\right) , \end{aligned}$$

since in the simultaneous limit $\delta \rightarrow 0$ and $\mu _0 \rightarrow \infty$, $\phi _0$ approaches $v_0$. The IR brane equation can then be used to separately determine $\delta$, and the result will be of the form

4.12 $$\begin{aligned} \delta ^4 = \chi ^4 f_1 (\phi _0, v_1,\lambda _1). \end{aligned}$$

Combining these two equations we find that the structure of the solutions to the scalar BCs will be of the form

4.13 $$\begin{aligned} \phi _0&= v_0 \left( 1 + \mathcal{O}( \chi ^4/\mu _0^4 ) \right) , \end{aligned}$$

4.14 $$\begin{aligned} \delta ^4&= \chi ^4 f_1 \left( v_0 ( 1 + \mathcal{O}( \chi ^4/\mu _0^4 ) ), \lambda _1, v_1\right) . \end{aligned}$$

These expressions have the right limits to be identified with an external source and a condensate:

4.15 $$\begin{aligned}&\lim _{\mu _0 \rightarrow \infty } \phi _0 = v_0,\end{aligned}$$

4.16 $$\begin{aligned}&\lim _{\chi \rightarrow 0} \delta ^4 = 0. \end{aligned}$$

For example in the limit $\lambda _{0,1}\rightarrow \infty$ we find

4.17 $$\begin{aligned} \begin{aligned} \phi _0&=v_0 +\frac{\sqrt{3}}{2\kappa } \log \left( \sqrt{1+\frac{\delta ^8}{\mu _0^8}}-\frac{\delta ^4}{\mu _0^4} \right) ,\\ \delta ^4&=\chi ^4 \sinh \left( \frac{2\kappa }{\sqrt{3}}(v_1-\phi _0) \right) , \end{aligned} \end{aligned}$$

and the system can be exactly solved, although the exact expressions are not important for the general argument.

The full effective dilaton potential is

4.18 $$\begin{aligned} V_\mathrm{eff} = V_\mathrm{UV} + V_\mathrm{IR} \end{aligned}$$

with

4.19 $$\begin{aligned} V_\mathrm{UV}&= \mu _0^4 \left[ \Lambda _0 -\frac{6k}{\kappa ^2} \sqrt{1+\frac{\delta ^8}{\mu _0^8}} + \lambda _0 \left( \phi _0-v_0\right. \right. \nonumber \\&\left. \left. -\frac{\sqrt{3}}{2\kappa }\log \left[ \sqrt{1+\frac{\delta ^8}{\mu _0^8}}-\frac{\delta ^4}{\mu _0^4}\right] \right) ^2 \right] , \end{aligned}$$

4.20 $$\begin{aligned} V_\mathrm{IR}&= \chi ^4 \left[ \Lambda _1 +\frac{6k}{\kappa ^2} \sqrt{1+\frac{\delta ^8}{\chi ^8}} + \lambda _1 \left( \phi _0-v_1\right. \right. \nonumber \\&\left. \left. -\frac{\sqrt{3}}{2\kappa }\log \left[ \sqrt{1+\frac{\delta ^8}{\chi ^8}}-\frac{\delta ^4}{\chi ^4}\right] \right) ^2 \right] . \end{aligned}$$

We can see that using (4.14) the IR term will become a pure quartic modulo the $\chi$-dependence of $\phi _0$, which is suppressed by the location of the UV brane, while the UV contribution will be a pure cosmological constant given by the RS tuning and additional $\chi ^4/\mu _0^4$-type corrections:

4.21 $$\begin{aligned}&V_\mathrm{IR} = \chi ^4 \left( a(v_0) +\mathcal{O}( \chi ^4/\mu _0^4) \right) ,\end{aligned}$$

4.22 $$\begin{aligned}&V_\mathrm{UV} = \mu _0^4 \left( \Delta _0 + \mathcal{O}( \chi ^8/\mu _0^8) \right) , \end{aligned}$$

where $a (v_0)$ is a constant that determines the quartic dilaton coupling, which depends on the UV value of the scalar field $v_0$ (and all the other parameters of the theory), while $\Delta _0$ is the usual RS UV fine tuning condition $\Delta _0 = \Lambda _0-6k/\kappa ^2$. For generic values of the parameters this potential would be minimized for $\chi \sim O(\mu _0)$ and thus no hierarchy would be generated.

Again for the sake of illustration, in the limit $\lambda _{0,1}\rightarrow \infty$ one finds the potentials

4.23 $$\begin{aligned}&V_\mathrm{UV}= \mu _0^4 \left[ \Lambda _0 - \frac{6k}{\kappa ^2} \right. \nonumber \\&\quad \quad \quad \quad \left. \left( 1+ \frac{\chi ^8 \sinh ^2 \left( \frac{2\kappa }{\sqrt{3}}(v_1-v_0) \right) }{\mu _0^8+\chi ^8-2\mu _0^4\chi ^4\cosh \left( \frac{2\kappa }{\sqrt{3}}(v_1-v_0) \right) } \right) ^{1/2} \right] ,\nonumber \\\end{aligned}$$

4.24 $$\begin{aligned}&V_\mathrm{IR}= -V_\mathrm{UV} (\mu _0 \leftrightarrow \chi , \Lambda _0 \rightarrow -\Lambda _1), \end{aligned}$$

and therefore the quartic dilaton coupling reads

4.25 $$\begin{aligned} a(v_0)= \Lambda _1 + \frac{6k}{\kappa ^2} \cosh \left( \frac{2\kappa }{\sqrt{3}}(v_1-v_0) \right) . \end{aligned}$$

This can be made to vanish by properly tuning the UV value of the scalar, $v_0$, which is the holographic equivalent to a tuning of the initial value of the external perturbation, $\lambda (\mu _0) \mathcal {O}$. It is particularly illuminating to notice that in the limit $\lambda _1 \rightarrow \infty$ we have taken, the whole IR potential comes from the $(6/\kappa ^2)A'$ piece, that is, from the back-reaction on the metric. This is easy to understand since the IR $\phi$ BC fixes $\phi ' \sim \partial V_1/\partial \phi$ and due to the structure of $V_1$ one has $V_1 \sim \phi ^{\prime 2}/\lambda _1 \rightarrow 0$ when $\lambda _1 \rightarrow \infty$.

The generic structure of the effective potential has a very clear explanation: the only explicit breaking of scale invariance in this theory corresponds to the introduction of the UV brane. Thus in the limit when the UV brane is removed, the effective potential must reduce to a pure quartic (plus a UV contribution to the cosmological constant). This is indeed what we find here, and the explicit expression for the quartic depends on $v_0$, the value of the scalar field in the UV. One can make the entire potential vanish by tuning the UV cosmological constant to zero, and by tuning $v_0$ appropriately. The important difference in this tuning compared to Goldberger–Wise is that here we tune the UV value of the scalar field (that is, the value of the perturbing coupling in the UV), rather than the IR brane tension (which is arbitrary here). We will see in the next section that this tuning will be alleviated once we let the perturbing coupling run, that is, once we include a non-trivial potential for $\phi$, in particular a mass term, $m^2 \sim \epsilon k^2$. Then $v_0 \rightarrow v_0 (\chi /\mu _0)^\epsilon$, which will become the leading order term in $\chi /\mu _0$ and will then set the hierarchy.

We should stress that once the tuning on $v_0$ is imposed corresponding to setting the quartic to zero, $a(v_0)=0$, the spacetime (3.2) with the warp factor given by (4.3) still represents the 5D dual of a spontaneously broken CFT, even though the metric deviates significantly from AdS:

4.26 $$\begin{aligned} \hbox {d}s^2= \sqrt{ \frac{\sinh 4k(y_\mathrm{c}-y)}{\sinh 4k y_\mathrm{c}}}\,\hbox {d}x^2 -\hbox {d}y^2. \end{aligned}$$

That this metric corresponds to a spontaneously broken scale invariant theory should be clear from the previous analysis and the resulting effective potential for the dilaton, but one can also explicitly consider the effect of the scale transformation $y\rightarrow y+a$, $x\rightarrow \hbox {e}^\alpha (a) x$. If the IR brane is kept fixed, then this transformation will not leave the metric invariant simply due to the presence of the IR brane^2^—this is exactly what one expects from a spontaneous breaking of scale invariance. The symmetry is restored by simultaneously moving the IR brane, $y_1\rightarrow y_1+a$. Due to the scalar BCs that result in (4.14) a shift in $y_1$ should also be accompanied by a shift in $y_\mathrm{c}$, which will make the shift in the warp factor $y$-independent: the net shift in the warp factor is then compensated by the scale factor $\hbox {e}^\alpha (a)=[\sinh (4k y_\mathrm{c})/\sinh (4k(y_\mathrm{c}+a))]^{1/2}$.^3^

Notice that in order to obtain a small cosmological constant (neglecting $O(\chi ^8/\mu _0^4)$ terms), we have to impose the UV RS tuning $\Delta _0 \ll 1$. This condition is actually also needed in order to obtain a suitable dilaton potential, due to the presence of a dilaton–gravity kinetic mixing, of $O(\chi ^2/\mu _0^2)$ (see Appendix E). If the UV RS tuning is not imposed we generate a term $\Delta _0 \mu _0^2 \chi ^2$ in the potential, which would not allow for the generation of a large hierarchy between $\mu _0$ and $\chi$.

In two appendices, Appendix B and C, we present the detailed description of the cases with a small back-reaction and with no bulk mass, and small back-reaction and small bulk mass (the GW case).

## Light dilaton without tuning: the general case

We are now ready to consider the general case with $O(1)$ IR brane mistuning, a large condensate and long slow running of the scalar due to a small scalar bulk mass. The bulk scalar potential is again given by

5.1 $$\begin{aligned} V(\phi ) = -\frac{6k^2}{\kappa ^2} - 2 \epsilon k^2 \phi ^2. \end{aligned}$$

We want to stress again that the exact form of the perturbing bulk potential does not matter, as long as it is always parametrically suppressed (that is, $\epsilon$ multiplies the entire bulk potential). For more complicated potentials the form of the RGE running will change, but as long as the $\epsilon$ suppression persists, the running will be mild. CPR suggested that the overall suppression of the bulk potential by $\epsilon$ may be due to $\phi$ being a 5D bulk Goldstone field, and $\epsilon$ is the parameter of a small explicit breaking term.

For the brane potentials we will again use a quadratic expression, Eq. (4.2), but as explained before the detailed form of this potential again does not matter.

In order to find the bulk solution, we note that we can break up the bulk into two regions: the UV region dominated by a mild RGE running of the scalar where the solution remains close to AdS, and the IR region dominated by the condensate, where the solution is of the form considered in the previous section. We will then match up these two solutions using asymptotic matching for the boundary layer theory of differential equations [31].

The UV solution is characterized by a mild running of the scalar, which means that one can neglect the second derivative of the scalar: $\phi ', \delta V(\phi ) \gg \phi ''$. The deviation from AdS space is small, so in this region $A' =k$, and the scalar equation is first order:

5.2 $$\begin{aligned} k \phi ' - \epsilon \phi =0, \end{aligned}$$

so the solution in the UV region (which we call the ‘running region’ and denote by subscript $r$) is given by

5.3 $$\begin{aligned} A'_r(y)&= k ,\end{aligned}$$

5.4 $$\begin{aligned} \phi _r(y)&= \phi _0 \hbox {e}^{\epsilon k y}. \end{aligned}$$

This solution is self-consistent in the UV as long as the back-reaction on the metric is negligible, that is, $\kappa ^2 \epsilon k^2 \phi ^2 /3 \ll A^{\prime 2}$, which restricts the region of validity to

5.5 $$\begin{aligned} y \ll \frac{1}{\epsilon k} \log \left( \frac{1}{\sqrt{\epsilon }\phi _0 \kappa }\right) . \end{aligned}$$

The second region where we can find an analytic solution is the region where the condensate dominates. In this case the behavior of the scalar is dominated by the $\phi '',\phi '$ terms and the additional bulk potential is negligible. In this case we recover the equations for the zero bulk mass considered in the previous section. Thus there is a universality in the IR behavior of the solution, since it is dominated by the dimension 4 condensate. Therefore in this IR ‘condensate region’ (denoted by the subscript c) the solution is given by

5.6 $$\begin{aligned} A'_\mathrm{c}(y)&= -k \coth \left( 4k(y-y_\mathrm{c}) \right) ,\end{aligned}$$

5.7 $$\begin{aligned} \phi _\mathrm{c}(y)&= \phi _\mathrm{m} - \frac{\sqrt{3}}{2 \kappa } \log \left( -\tanh \left( 2k(y-y_\mathrm{c}) \right) \right) , \end{aligned}$$

where $\phi _\mathrm{m}$ is the matching value of the scalar field. Applying the method of asymptotic matching for a boundary layer theory we obtain the matching conditions:

5.8 $$\begin{aligned} \lim _{y \rightarrow -\infty } \phi _\mathrm{c} = \lim _{y \rightarrow y_1} \phi _\mathrm{r}&\Rightarrow \phi _\mathrm{m} = \phi _0 \hbox {e}^{\epsilon k y_1},\end{aligned}$$

5.9 $$\begin{aligned} \lim _{y \rightarrow -\infty } A'_\mathrm{c} = \lim _{y \rightarrow y_1} A'_\mathrm{r}&\Rightarrow k=k. \end{aligned}$$

The details of this matching are explained in Appendix D.

As before, to determine the constants $\phi _0$ and $y_\mathrm{c}$ we impose the UV BC for $\phi _\mathrm{r}$ and the IR BC for $\phi _\mathrm{c}$:

5.10 $$\begin{aligned} 2\phi '_\mathrm{r} |_{y=y_0}&= + \frac{\partial V_{0}}{\partial \phi }\Bigg |_{\phi (y)=\phi _\mathrm{r}(y_0),} \end{aligned}$$

5.11 $$\begin{aligned} 2\phi '_\mathrm{c}|_{y=y_1}&= \!-\! \frac{\partial V_{1}}{\partial \phi }\Bigg |_{\phi (y)\!=\!\phi _\mathrm{c}(y_1),} \quad \end{aligned}$$

from which we find, in the limit $\lambda _0, \lambda _1 \rightarrow \infty$,

5.12 $$\begin{aligned}&\phi _0 = v_0 \mu _0^{\epsilon },\end{aligned}$$

5.13 $$\begin{aligned}&\delta = \chi \tanh ^{1/4} \left( \frac{\kappa }{\sqrt{3}}(v_1-\phi _\mathrm{m}) \right) . \end{aligned}$$

To simplify our expressions we have used the alternate definition of the dilaton, the UV scale, and the condensate $\mu _0 = \hbox {e}^{-ky_0}$, $\delta = \hbox {e}^{-ky_\mathrm{c}}$, and $\chi = \hbox {e}^{-ky_1}$. As we learned from the constant bulk potential case, the distance between the singularity and the IR brane, or equivalently $\delta /\chi$, depends on the IR potential parameters, in particular on the difference between $\phi (y_1) = v_1$, and $\phi (y_0) = v_0$, where the latter is now modulated by $(\mu _0/\chi )^{\epsilon }$.

The full approximate solution^4^ to the system is

5.14 $$\begin{aligned} \phi _\mathrm{full}(y)&= \phi _\mathrm{r}(y) + \phi _\mathrm{c}(y) - \phi _\mathrm{m} \end{aligned}$$

5.15 $$\begin{aligned}&= v_0 \, \hbox {e}^{\epsilon k (y-y_0)} - \frac{\sqrt{3}}{2 \kappa } \log \left( \!\tanh \left( 2k(y_\mathrm{c}\!-\!y) \right) \right) \quad \end{aligned}$$

and equivalently for $A'(y)$. In $z=\hbox {e}^{-ky}$ coordinates these are

5.16 $$\begin{aligned}&A'_\mathrm{full}(z)\!=\!\left( \!\!-\!1 \!+\! \frac{2 z^8}{z^8 \!+\! \chi ^8 \tanh ^2 \left( \!\frac{\kappa }{\sqrt{3}}(\!v_1\!-\!v_0 (\mu _0/\chi )^{\epsilon }) \right) } \right) ^{-1} ,\nonumber \\ \end{aligned}$$

5.17 $$\begin{aligned}&\phi _\mathrm{full}(z)= v_0 \left( \frac{\mu _0}{z} \right) ^{\epsilon } - \frac{\sqrt{3}}{2 \kappa } \nonumber \\&\quad \quad \quad \!\!\quad \quad \quad \log \left[ -1\!+\! \frac{2 z^4}{z^4 \!+\! \chi ^4 \tanh \left( \frac{\kappa }{\sqrt{3}}(v_1\!-\!v_0 (\mu _0/\chi )^{\epsilon })\right) } \right] \! .\nonumber \\ \end{aligned}$$

**Fig. 2:**
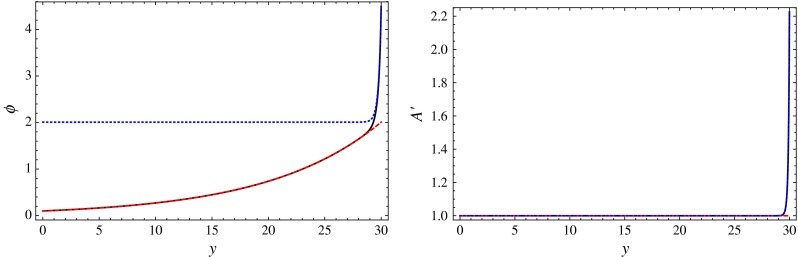
*Left* Bulk scalar profile: $\phi _\mathrm{full}$ (*solid black*), $\phi _\mathrm{r}$ (*dashed red*), and $\phi _\mathrm{b}$ (*dotted blue*). *Right* Effective AdS curvature, $A'(y)$: same color code

This solution exhibits the correct asymptotic behavior. We can see this explicitly in Fig. 2. The full solution interpolates nicely between the running and the condensate-dominated solutions.

We can now compute the effective potential for the dilaton as usual (again in the $\lambda _{0,1}\rightarrow \infty$ limit)

5.18 $$\begin{aligned} V_\mathrm{UV}&= \mu _0^4 \left[ \Lambda _0 -\frac{6k}{\kappa ^2} \right] ,\end{aligned}$$

5.19 $$\begin{aligned} V_\mathrm{IR}&= \chi ^4 \left[ \Lambda _1 + \frac{6 k}{\kappa ^2} \cosh \left( \frac{2 \kappa }{\sqrt{3}} (v_1 - v_0 (\mu _0/\chi )^{\epsilon }) \right) \right] \nonumber \\&\mathrm{sech}^2 \left( \frac{\kappa }{\sqrt{3}} (v_1 - v_0 (\mu _0/\chi )^{\epsilon } )\right) . \end{aligned}$$

The UV effective potential contains a constant piece, which must be tuned to zero in order to obtain a flat 4D space (usual UV RS tuning). The IR potential is of the expected form $\chi ^4 F[(\mu _0/\chi )^{\epsilon }]$. This is the leading part of the potential, whose minimization will determine the position of the minimum, $\langle {\chi } \rangle$, up to $O(\epsilon )$ corrections. Recall also that the potentials Eqs. (5.18) and (5.19) are corrected by $O(\chi ^2/\mu _0^2)$ once the dilaton–gravity kinetic mixing is fully included, see Appendix E. It is therefore important to tune $\Lambda _0 \simeq 6 k / \kappa ^2$ in order not to generate a large $\chi ^2$ term.

To leading order in $\epsilon$, the condition for the minimum of the potential is

5.20 $$\begin{aligned} \frac{\partial V_\mathrm{IR}}{\partial \chi } = \chi ^3 \left( 4 F[(\mu _0/\chi )^\epsilon ] + F'[(\mu _0/\chi )^\epsilon ] \epsilon (\mu _0/\chi )^{\epsilon } \right) \!=\! 0,\nonumber \\ \end{aligned}$$

leading to a dilaton VEV

**Fig. 3:**
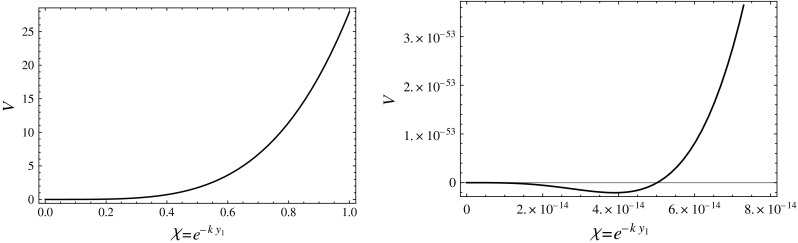
The plot of the effective dilaton potential Eq. (5.19) for the parameters $\epsilon =0.1$, $v_0=0.1$, $v_1=4.5$, $\Lambda _1 = - 50$, $\mu _0 = 1$, and $\kappa = 0.5$, all of them in units $k=1$. The plot in the *right* is a zoom of the region where the minimum of the potential is

5.21 $$\begin{aligned} \frac{\langle {\chi } \rangle }{\mu _0}&= \left( \frac{v_0}{v_1 \!- \, \mathrm{sign}(\epsilon ) \frac{\sqrt{3}}{2 \kappa } \, \mathrm{arcsech} (-6k/\kappa ^2 \Lambda _1)} \right) ^{1/\epsilon }\!+\! O(\epsilon ),\nonumber \\ \end{aligned}$$

while the potential will obviously be of order $F[(\mu _0/\chi )^\epsilon ] = \mathcal{O}(\epsilon )$. Notice that for this to be a good minimum we need $\Lambda _1 < 0$ and $|\Lambda _1| > 6k/\kappa ^2$. One can clearly see from Eq. (5.19) that if these conditions are not satisfied then the effective quartic is always positive $F[\chi /\mu _0]> 0$ for all $\chi$, and the minima can only be found at $\langle {\chi } \rangle =0$ or $\langle {\chi } \rangle =\mu _0$. Furthermore, in order for the effective quartic to be positive at $\chi = \mu _0$ (thus avoiding this as a minimum), one must have $|\Lambda _1| < \frac{6k}{\kappa ^2} \cosh ( \frac{2 \kappa }{\sqrt{3}} (v_1 - v_0) )$. This condition is easily satisfied, either if $v_1 \gg v_0$, a condition consistent with $\epsilon > 0$, or $v_0 \gg v_1$, consistent with $\epsilon < 0$. However, notice that a large hierarchy, which in this scenario is given by the point where $6A'/\kappa ^2$ compensates $\Lambda _1$, is easier to produce for the case $\epsilon > 0$, since in this case $v_1 - v_0(\mu _0/\chi )^\epsilon$ runs slower than for $\epsilon <0$. This is the scenario we have advocated for naturally canceling a large quartic at the scale $\mu _0$. We show a plot of the potential (5.19) in Fig. 3, where we can see that a shallow stable minimum with a small mass is indeed generated.

One might be concerned that phase transitions in the early universe may substantially affect the Planck-weak hierarchy, effectively changing $G_N$. However, the sensitivity to changes in $\Lambda _1$ from condensates is only power-law:

5.22 $$\begin{aligned}&\left| \frac{\partial \log \chi }{\partial \log \Lambda _1}\right| =\frac{1}{\epsilon }\frac{\sqrt{3}}{2\kappa v_0\sqrt{1-\left( \frac{6k}{\kappa ^2\Lambda _1}\right) ^2}}\left( \frac{\chi }{\mu _0}\right) ^{\epsilon }\,. \end{aligned}$$

For example, a change in the IR tension due to the QCD phase transition would give rise to $\delta \Lambda _1/\Lambda _1\sim (\Lambda _\mathrm{QCD}/\hbox {TeV})^{4} \sim 10^{-16}$ and hence it generates only a small change in the hierarchy $\delta \chi /\chi \propto \frac{1}{\epsilon }\delta \Lambda _1/\Lambda _1$ as long as $\epsilon$ is bigger than $\sim 10^{-14}$.

The dual CFT interpretation of the potential Eq. (5.19) for the interesting $\epsilon > 0$ is simple. The quartic in the absence of perturbation (that is, $v_0 = 0$) is given by $F_0 = \Lambda _1 + \frac{6k}{\kappa ^2} \cosh (\frac{2 \kappa }{\sqrt{3}}v_1)$. This is generically large and positive, hence there is no SBSI at high scales. Once the perturbation is turned on, it grows larger in the IR, $v_0 (\mu _0/\chi )^{\epsilon }$. This in turn decreases the effective quartic, until the minimum $F[\chi /\mu _0]={\mathcal O}(\epsilon )$ is found. Effectively, the dilaton quartic coupling relaxes to zero at $\chi /\mu _0 \ll 1$. At this point SBSI will occur.

The dilaton mass to leading order in $\epsilon$ is given by

5.23 $$\begin{aligned} m_\chi ^2&\sim \epsilon \frac{32 \sqrt{3} k v_0}{\kappa } \tanh \left( \frac{\kappa }{\sqrt{3}}(v_1 - v_0(\mu _0/\chi )^{\epsilon }) \right) \nonumber \\&\langle {\chi } \rangle ^2 (\mu _0/\chi )^{\epsilon } + O(\epsilon ^2). \end{aligned}$$

One then concludes that, regardless of the size of the back-reaction on the metric, the dilaton remains light as long as the $\beta$-function is small. Of course the actual physical mass of the dilaton also depends on the normalization of its kinetic term, which we have not calculated in this paper, we assume it is $O(1)$ or bigger. The kinetic term normalization does not remove the $\epsilon$ suppression in (5.23).

Next we examine the value of the potential at the minimum, which is the effective cosmological constant. In the approximation we have followed in this section, the cosmological constant is given by $\Lambda _\mathrm{eff}=V_\mathrm{IR}(\langle {\chi } \rangle )$ from Eq. (5.19), since we have fine-tuned away $V_\mathrm{UV}$. The value of the IR potential at the minimum is

5.24 $$\begin{aligned} V_\mathrm{IR}^\mathrm{min}&= - \epsilon \frac{2 \sqrt{3} k v_0}{\kappa } \tanh \left( \frac{\kappa }{\sqrt{3}}(v_1 - v_0(\mu _0/\chi )^{\epsilon }) \right) \nonumber \\&\langle {\chi } \rangle ^4 (\mu _0/\chi )^{\epsilon } \sim -m_{\chi }^2 \frac{\langle {\chi } \rangle ^2}{16}. \end{aligned}$$

As expected, the value of the minimum is suppressed by $\epsilon$, and also by $4-\epsilon$ powers of the dilaton at the minimum. Assuming that this is the origin of the hierarchy, that is, $\langle \chi \rangle k \sim$ TeV, the resulting potential is of order $\epsilon$ TeV$^4$. Therefore, since we have minimized the potential at $O(\epsilon ^0)$ Eq. (5.20), $V_\mathrm{IR}(\langle {\chi } \rangle ) = O(\epsilon \langle {\chi } \rangle ^4 k^4)$. Phenomenologically, this contribution is still too large unless $\epsilon \sim 10^{-60}$, which, however, would require much more running than available from the observed Planck-weak hierarchy, and which would also yield a dilaton that is too light, $\sim \!10^{-18}$ eV. Also, since $\epsilon > 0$ for most interesting applications, the IR potential is usually negative. Of course since our full potential contained a tuned value of the UV contribution, one could have tuned $V_\mathrm{UV} = O(\epsilon \langle {\chi } \rangle ^4 k^4)$ previously, such that eventually $\Lambda _\mathrm{eff} = 0$ or small positive. This change in the UV potential affects the minimization only at $O(\epsilon )$, and thus it does not affect our conclusions.

We finally show that regardless of the explicit form of the IR brane potential the value of the potential at the minimum is always suppressed by $\epsilon$. The form of the dilaton potential is $\hbox {e}^{-4A(y_1)} F(y_1,y_\mathrm{c})$, hence the derivative of the potential is given by

5.25 $$\begin{aligned} \frac{\partial V_\mathrm{IR} }{\partial y}\Bigg |_{y_1}&= \hbox {e}^{-4 A(y)} \left( -4 A'(y) F(y,y_\mathrm{c}) \right. \nonumber \\&\!\left. + \frac{\hbox {d}}{\hbox {d}y} F(y,y_\mathrm{c}) \!+\!\frac{\hbox {d}}{\hbox {d}y_\mathrm{c}} F(y,y_\mathrm{c}) \frac{\hbox {d}y_\mathrm{c}}{\hbox {d}y_1} \right) \Bigg |_{y_1} \!=\! 0.\nonumber \\ \end{aligned}$$

Note that

5.26 $$\begin{aligned} \frac{\hbox {d}}{\hbox {d}y} F(y,y_\mathrm{c})= \frac{\partial V_1}{\partial \phi } \phi '+\frac{6}{\kappa ^2} A^{\prime \prime }, \end{aligned}$$

and by using the bulk equation of motion $A^{\prime \prime } = \kappa ^2 \phi ^{\prime 2}/3$ can be brought to a form proportional to the scalar boundary condition (3.7), and thus it vanishes at the IR brane. Note also that the functional dependence of $F$ on $y_\mathrm{c}$ comes in the form $y_\mathrm{c} -y$, so in the $\epsilon \rightarrow 0$ limit we also have $\frac{\hbox {d}}{\hbox {d}y_\mathrm{c}} F(y,y_\mathrm{c})=0$. Thus at the minimum

5.27 $$\begin{aligned} \frac{\hbox {d}}{\hbox {d}y_\mathrm{c}} F(y,y_\mathrm{c})&= -\frac{\hbox {d}}{\hbox {d}y} F(y,y_\mathrm{c}) +\epsilon \, k \, \phi _0 \,\hbox {e}^{\epsilon k (y-y_0) }\frac{\partial V_1}{\partial \phi }\nonumber \\&= \epsilon \, k \, \phi _0 \,\hbox {e}^{\epsilon k (y-y_0) }\frac{\partial V_1}{\partial \phi }; \end{aligned}$$

thus for the value of the potential at the minimum we find

5.28 $$\begin{aligned} F|_\mathrm{min}= \frac{ \epsilon \, k \, \phi _0 \,\hbox {e}^{\epsilon k (y_1-y_0) }}{4A'(y_1)} \frac{\partial V_1}{\partial \phi } \frac{\hbox {d}y_\mathrm{c}}{\hbox {d}y_1}. \end{aligned}$$

## Conclusions

We presented a 5D holographic construction of a theory with a naturally light dilaton: a conformal theory perturbed by an almost marginal (dimension $4-\epsilon$) operator. As the coupling of the perturbation slowly increases through renormalization group running, the effective quartic of the dilaton slowly decreases. Around the scale where the effective quartic vanishes scale invariance will be broken, the perturbing operator (along with other CFT operators) will develop a condensate and we have a stable minimum of the dilaton potential at hierarchically small scales. If the perturbing operator remains close to marginal even for large couplings, the dilaton mass squared and the value of the dilaton potential at the minimum will both be suppressed by $\epsilon$.

In order to find the explicit holographic description of this setup we first considered the case with an exactly marginal perturbation, and described the exact solutions of the scalar-gravity equations for this system. This solution is a novel holographic dual of an exactly conformal theory where conformality is broken via the condensate of a dimension 4 operator. Even though the metric deviates significantly from AdS in the IR, this nevertheless corresponds to a non-linearly realized conformal theory. This solution provides the description of the IR region for the case with the $4-\epsilon$-dimensional condensate, while the UV is dominated by the slow running of the bulk scalar. Matching these solutions one obtains the full background for the system with the light dilaton. Finally we applied the formula for the effective dilaton potential derived earlier in this paper to verify that the dilaton mass squared and the contribution to the cosmological constant are both indeed suppressed by $\epsilon$. Phenomenologically $\epsilon$ cannot be taken small enough to solve the cosmological constant problem: an exponentially small $\epsilon$ requires much more running than the observed Planck-weak hierarchy would suggest. However, a parametric suppression over the value in SUSY is still interesting (and as far as we know unique to this mechanism).

## Appendix A: Effective potential and boundary terms

We show here another way to get the effective dilaton potential (3.12) from integrating out the extra dimension. In order to properly disentangle brane and bulk contributions to $V_\mathrm{eff}(\chi )$ it is convenient to explicitly write the Gibbons–Hawking boundary terms,

7.1 $$\begin{aligned} S&= S_\mathrm{bulk}-\int \hbox {d}^4x \sqrt{g_0} V_0 (\phi ) - \int \hbox {d}^4x \sqrt{g_1} V_1 (\phi )\nonumber \\&-\frac{1}{\kappa ^2}\int \hbox {d}^4x\sqrt{g_0} K_0 -\frac{1}{\kappa ^2}\int \hbox {d}^4x\sqrt{g_1} K_1 \end{aligned}$$

where $K_{0,1}$ are the extrinsic UV and IR curvatures that for a rigid brane $y=y_{0,1}$ in our warped metric are $K_{0,1}=\nabla _M n^M=\mp 4A^\prime (y_{0,1})$. $n^M=(0,\pm )$ is the normal unit vector and $-$ (+) is for the UV (IR) brane. This contribution gets actually doubled, because of the orbifolding, after integrating out the extra dimension,

7.2 $$\begin{aligned} V^\mathrm{boundary}_\mathrm{UV/IR}=\hbox {e}^{-4A(y_{0,1})}\left[ V_{0,1}(\phi )\mp \frac{8}{\kappa ^2} A^{\prime }(y_{0,1})\right] , \end{aligned}$$

and it adds to the bulk contribution $V_\mathrm{bulk}=\pm 2/\kappa ^2 A^\prime (y_{0,1})$ (after using the $\phi$ equations of motion)

7.3 $$\begin{aligned} V_\mathrm{UV/IR}=\hbox {e}^{-4A(y_{0,1})}\left[ V_{0,1}(\phi )\mp \frac{6}{\kappa ^2} A^{\prime }(y_{0,1})\right] , \end{aligned}$$

giving the effective potential (3.12).

## Appendix B: The massless case for small back-reaction

The computation of the effective potential in Sect. 4 can be explicitly carried out for the case when the back-reaction of the metric is small, that is, when we expand around the usual AdS solution ($\delta /\chi \ll 1$). In this case the expanded bulk solutions are

8.1 $$\begin{aligned} A(y)&= ky + \frac{\delta ^8}{16} \left( \hbox {e}^{8ky}-1 \right) + O(\delta ^9) ,\end{aligned}$$

8.2 $$\begin{aligned} \phi (y)&= \phi _0 + \frac{\sqrt{3}}{2 \kappa } \delta ^4 \hbox {e}^{4ky} + O(\delta ^9). \end{aligned}$$

Solving the scalar BCs Eq. (3.7) for $\phi _0$ and $\delta$ at $O(\delta ^8)$, with the brane potentials Eq. (4.2), we find

8.3 $$\begin{aligned} \phi _0&= \frac{v_1 (4 k - \lambda _0) \lambda _1 \chi ^4 + v_0 (4 k + \lambda _1) \lambda _0 \mu _0^4}{(4 k - \lambda _0) \lambda _1 \chi ^4 + (4 k + \lambda _1) \lambda _0 \mu _0^4},\end{aligned}$$

8.4 $$\begin{aligned} \delta ^4&= \frac{2 \kappa }{\sqrt{3}} (v_1 - v_0) \frac{\lambda _0 \lambda _1 \mu _0^4 \chi ^4}{\mu _0^4 (4k + \lambda _1) \lambda _0 + \chi ^4 (4 k - \lambda _0) \lambda _1} .\nonumber \\ \end{aligned}$$

For the effective potential we find at leading order in $\chi /\mu _0$

8.5 $$\begin{aligned}&V_\mathrm{UV} = \Delta _0 \mu _0^4 + O(\chi ^8/\mu _0^4),\end{aligned}$$

8.6 $$\begin{aligned}&V_\mathrm{IR} = \left( \Delta _1 + \frac{4 k (v_0-v_1)^2 \lambda _1}{4k + \lambda _1} \right) \chi ^4 + O(\chi ^8/\mu _0^4) \end{aligned}$$

where $\Delta _0 = \Lambda _0 - 6k/\kappa ^2$ and $\Delta _1 = \Lambda _1 + 6k/\kappa ^2$, the mistunings of UV and IR brane tensions. These expressions have the expected generic structure of (4.21)–(4.22).

The rescaling of the potential after taking into account the dilaton–graviton mixing is given by (see Appendix E)

8.7 $$\begin{aligned} \frac{1}{K^2} = 1 + 2 \frac{\chi ^2}{\mu _0^2} \left( 1 +\frac{4 \kappa ^2 \lambda _1^2 (v_0-v_1)^2}{3 (4k+\lambda _1)^2} \right) + O(\chi ^4/\mu _0^4),\nonumber \\ \end{aligned}$$

verifying that it is $O(\chi ^2/\mu _0^2)$.

## Appendix C: (Mistuned) Goldberger–Wise revisited

We revisit here the scenario discussed in Sect. 5 under the assumption of a small mistune $\Delta _1$ and small back-reaction. This effectively corresponds to the analysis of GW supplemented by a small mistune ($\Delta _1\ll 1$).

The solutions to the bulk equations of motion close to AdS space, at $O(\delta ^8 \sim \kappa ^2 \phi ^2)$, and now also at $O(\epsilon )$ and at all orders in $\hbox {e}^{\epsilon ky}$, is given by

9.1 $$\begin{aligned} A(y)&\simeq ky + \frac{\delta ^8}{16} \left( \hbox {e}^{(8-2\epsilon ) ky}-1 \right) + \frac{\kappa ^2 \phi _0^2}{12} \left( \hbox {e}^{2\epsilon ky}-1 \right) \nonumber \\&+ \epsilon \frac{\delta ^4 \kappa \phi _0 \sqrt{3}}{12} \left( \hbox {e}^{4 ky}-1 \right) ,\end{aligned}$$

9.2 $$\begin{aligned} \phi (y)&\simeq \phi _0 \hbox {e}^{\epsilon k y} + \frac{\sqrt{3}}{2 \kappa } \delta ^4 \hbox {e}^{(4-\epsilon ) ky} \end{aligned}$$

where we have again fixed $A(0)=0$. This expansion ensures that we include in the effective potential all terms at a given order up to $O(\delta ^8 \sim \kappa ^2 \phi ^2)$, including also $O(\epsilon )$ terms, and at all orders in $\hbox {e}^{\epsilon ky}$.

Solving now the scalar BCs Eq. (3.7) for $\phi _0$ and $\delta$, with the brane potentials Eq. (4.2), we find at $O(\epsilon )$,

9.3 $$\begin{aligned} \phi _0&= \frac{v_1 (4 k - \lambda _0) \lambda _1 \chi ^{4-\epsilon } + v_0 (4 k + \lambda _1) \lambda _0 \mu _0^{4-\epsilon }}{(4 k - \lambda _0) \lambda _1 \chi ^{4-2\epsilon } + (4 k + \lambda _1) \lambda _0 \mu _0^{4-2\epsilon }}+ \epsilon (\dots ), \nonumber \\\end{aligned}$$

9.4 $$\begin{aligned} \delta ^4&\simeq \frac{2 \kappa }{\sqrt{3}} (\mu _0 \chi )^{4-\epsilon } \frac{\lambda _0 \lambda _1 (v_1 \mu _0^{-\epsilon } - v_0 \chi ^{-\epsilon })}{\!\mu _0^{4-2\epsilon } (4k \!+\! \lambda _1) \lambda _0 \!+\! \!\chi ^{4-2\epsilon } (\!4 k\! \!- \lambda _0) \lambda _1} \!+\! \epsilon (\dots )\nonumber \\ \end{aligned}$$

where we have omitted the $O(\epsilon )$ terms to avoid clutter. One can explicitly check that in the limit $\epsilon \rightarrow 0$ we recover Eqs. (8.3) and (8.4), furthermore in the limit $\lambda _0, \lambda _1 \rightarrow \infty$ limit we recover the well-known results of Rattazzi and Zaffaroni [6].

We can now compute the effective potential as in Eqs. (4.19) and (4.20), as a function of $\chi$, $\mu _0$, $\phi _0$, and $\delta$. The potential, using the expressions for $\phi _0$ and $\delta$ in Eqs. (9.3) and (9.4), at leading order in $\chi /\mu _0$ and in the limit $\lambda _0, \lambda _1 \rightarrow \infty$, is given by

9.5 $$\begin{aligned}&V_\mathrm{UV} = (\Delta _0 - \epsilon k v_0^2) \mu _0^4 - \epsilon 2 k v_0 (\mu _0/\chi )^{2\epsilon } \nonumber \\&( v_1-v_0 (\mu _0/\chi )^{\epsilon }) \chi ^4 + O(\chi ^8/\mu _0^4), \end{aligned}$$

9.6 $$\begin{aligned} V_\mathrm{IR}&= \left( \Delta _1 + 4 k (v_1-v_0(\mu _0/\chi )^{\epsilon })^2 + \epsilon (\dots ) \right) \chi ^4\nonumber \\&\left( 1-\frac{\kappa ^2(v_1-v_0(\mu _0/\chi )^{\epsilon })}{3} \right) + O(\chi ^8/\mu _0^4). \end{aligned}$$

This is in agreement with our expectations: the value of $v_0$ is replaced by the running coupling $v_0 (\mu _0/\chi )^\epsilon$, making the dilaton quartic effectively run, which will allow a non-trivial minimum of the potential as in [5, 6].

## Appendix D: Asymptotic matching

In order to perform the asymptotic matching between the running and the boundary solutions it is somewhat more convenient to change coordinates and rewrite the equations of motion (3.5) for $\Phi (y)\equiv \phi (y/\epsilon )$

10.1 $$\begin{aligned}&\epsilon \Phi ^{\prime \prime }(y)-4A^\prime (y/\epsilon )\, \Phi ^\prime (y)+4 k^2 \Phi (y) = 0,\end{aligned}$$

10.2 $$\begin{aligned}&A^{\prime \,2}(y/\epsilon )=\frac{\epsilon ^2\kappa ^2}{12}\Phi ^{\prime \,2}(y)+\frac{\epsilon \kappa ^2 k^2}{3}\Phi ^2(y)+k^2 \end{aligned}$$

where we have specified the bulk potential $V=-6k^2/\kappa ^2-2\epsilon k^2\phi ^2$. These equations, for $\epsilon \ll 1$, show a boundary layer close to the IR brane, and one can directly apply the boundary layer theory of [31]. In the outer (UV) region where $\Phi$ and $A$ are slowly varying we can neglect all the $O(\epsilon )$ terms so that the solution is well approximated by

10.3 $$\begin{aligned} \Phi _\mathrm{r}(y)=\Phi _0 \hbox {e}^{k y},\qquad A_\mathrm{r}^\prime =k \end{aligned}$$

where $\Phi _0$ is determined by the UV BC

10.4 $$\begin{aligned} \Phi _0=v_0 \mathrm{e}^{-k y_0}. \end{aligned}$$

As $\Phi$ approaches the boundary layer it starts running fast so that we can neglect the mass term in the potential but not necessarily the cosmological constant contribution that can still be large: the boundary solution is thus given by

10.5 $$\begin{aligned}&\Phi _\mathrm{b}(y) = \Phi _\mathrm{m} - \frac{\sqrt{3}}{2 \kappa } \log \left( -\tanh \left[ 2k/\epsilon (y-\tilde{y}_\mathrm{c}) \right] \right) ,\end{aligned}$$

10.6 $$\begin{aligned}&A^\prime _\mathrm{b}(y/\epsilon ) = -k \coth \left( 4k/\epsilon (y-\tilde{y}_\mathrm{c}) \right) \end{aligned}$$

where we have defined $\tilde{y}_\mathrm{c}=\epsilon y_\mathrm{c}$. The thickness of the boundary layer is determined by

10.7 $$\begin{aligned} y_\mathrm{b}-\tilde{y}_\mathrm{c}\sim \epsilon /2k \end{aligned}$$

where $A'_\mathrm{b}$ and $\Phi _\mathrm{b}$ start approaching a constant.

The IR BC fixes only one of the two integration constants; the other is going to be fixed by the asymptotic matching with the UV solution.

The asymptotic matching takes place at the edges of the inner and outer regions: in this overlap region both $\phi _\mathrm{r,b}$ are solutions. For this boundary region we can take for example $y-\tilde{y}_\mathrm{c}\sim \epsilon ^{1/2}/2k$ for $\epsilon \ll 1$

10.8 $$\begin{aligned} \Phi _0 \hbox {e}^{k \tilde{y}_\mathrm{c}}= \Phi _\mathrm{m}. \end{aligned}$$

Of course we can match in any other location as long it is in the overlapping region. It actually makes more sense to match at the branes at $y_1$ and $y_0$, which corresponds to taking $\epsilon$ small but finite,

10.9 $$\begin{aligned} \Phi _0 \hbox {e}^{k y_1}= \Phi _\mathrm{m}- \frac{\sqrt{3}}{2 \kappa } \log \left( -\tanh \left[ 2k/\epsilon (y_0-\tilde{y}_\mathrm{c}) \right] \right) . \end{aligned}$$

In this case the full solution is given by

10.10 $$\begin{aligned} \Phi&= \Phi _\mathrm{b}+\Phi _\mathrm{r}- \Phi _\mathrm{match}=\Phi _0 \hbox {e}^{k y}\nonumber \\&- \frac{\sqrt{3}}{2 \kappa } \log \left( \frac{\tanh \left[ 2k/\epsilon (\tilde{y}_\mathrm{c}-y) \right] }{\tanh \left[ 2k/\epsilon (\tilde{y}_\mathrm{c}-y_0) \right] } \right) \end{aligned}$$

where $\Phi _0$ and $\tilde{y}_\mathrm{c}$ in this expression are actually given in terms of $v_{0,1}$ and $y_{0,1}$ via the BCs.

Notice that the scale $y_\mathrm{br}$ where the back-reaction becomes important for the running solution is

10.11 $$\begin{aligned} y_\mathrm{br}\sim \frac{1}{k}\log \left( \sqrt{3}/(\epsilon ^{1/2}\kappa \Phi _0)\right) , \end{aligned}$$

which agrees with QCD where $\Lambda _\mathrm{QCD}$ is fixed by the UV coupling and its $\beta$-function: barring tuning, one expects $y_1\sim y_\mathrm{br}\sim y_\mathrm{c}$.

Transforming back to the original coordinates we obtain (5.15).

## Appendix E: Dilaton kinetic term

We can parametrize the fluctuations of the 5D metric of Eq. (3.2) as

11.1 $$\begin{aligned} \hbox {d}s^2 = \hbox {e}^{-2 W(x,y)} \hat{g}_{\mu \nu }(x)\,\hbox {d}x^\mu \,\hbox {d}x^\nu - T(x,y)^2\,\hbox {d}y^2. \end{aligned}$$

Upon evaluation of the 5D Ricci scalar term in Eq. (3.1) in terms of the metric Eq. (11.1), one obtains

11.2 $$\begin{aligned} \mathcal {L}_\mathrm{eff}^\mathrm{(kin)}&= -\frac{1}{\kappa ^2} \int _{y_0}^{y_1}\,\hbox {d}y \sqrt{g} \mathcal {R} \nonumber \\&= \frac{1}{\kappa ^2} \!\int _{y_0}^{y_1}\,\hbox {d}y \!\sqrt{\hat{g}} \hbox {e}^{- 2 W} \left( \!T \mathcal {R}[\hat{g}] \!-\! 6 T (\partial W)^2 \!+\! 6 \partial T \partial W\right) ,\nonumber \\ \end{aligned}$$

where we have dropped $\partial ^\mu \hat{g}_{\mu \nu }$ terms by gauge-fixing the gravity fluctuations to be transverse. The second line of Eq. (11.2) makes evident that the 5D Ricci scalar contains both dilaton interactions (kinetic mixing) with gravity via the first term in the parentheses, as well as the kinetic term for the dilaton, the second and third terms.

A proper parametrization to describe the dilaton fluctuations is given by Csaki et al. [10] (see also [32]),

11.3 $$\begin{aligned} W(x,y) = A(y) + F(x,y), \quad T(x,y) = 1 + 2 F(x,y) .\nonumber \\ \end{aligned}$$

Expanding Eq. (11.2) on the fluctuation, we obtain at quadratic order

11.4 $$\begin{aligned} \mathcal {L}_\mathrm{eff}^\mathrm{(kin)}&= \frac{1}{\kappa ^2} \int _{y_0}^{y_1} \hbox {d}y \sqrt{\hat{g}} \hbox {e}^{- 2 A(y)}\nonumber \\&\left[ (1+2F^2) \mathcal {R}[\hat{g}]+ 6 (\partial F)^2 + O(F^3) \right] . \end{aligned}$$

An ansatz for the case where we can neglect the fluctuations of the the bulk scalar, in particular for the limit $\lambda _0, \lambda _1 \rightarrow \infty$ in Eq. (4.2), is given by $F(x,y) = f(x) \hbox {e}^{2A(y)}/\hbox {e}^{2A(y_1)}$. In this case one obtains

11.5 $$\begin{aligned} \mathcal {L}_\mathrm{eff}^\mathrm{(kin)}&= \frac{1}{\kappa ^2} \int _{y_0}^{y_1} \hbox {d}y \sqrt{\hat{g}} \left[ \left( \hbox {e}^{- 2 A(y)}+2f^2 \frac{\hbox {e}^{2 A(y)}}{\hbox {e}^{2A(y_1)}}\right) \mathcal {R}[\hat{g}]\right. \nonumber \\&\left. + 6 \frac{\hbox {e}^{2 A(y)}}{\hbox {e}^{2A(y_1)}} (\partial f)^2 + O(f^3) \right] . \end{aligned}$$

### E.1 Dilaton reparametrizations

In the main text we have parametrized the background dilaton solution by $\chi =\hbox {e}^{-A(y_1)}$ or in some cases it was more convenient to use a different parametrization $\tilde{\chi } = \hbox {e}^{-ky_1}$. Notice from Eq. (3.12) that the latter does not yield automatically a quartic dilaton potential when the metric is not AdS. In fact the relation between the two parametrizations can be explicitly computed for the metric Eq. (4.3),

11.6 $$\begin{aligned} \chi ^4&= \frac{2 \tilde{\chi }^4}{1+\tilde{\chi }^8 + (1-\tilde{\chi }^8) \cosh \left( \frac{2 \kappa }{\sqrt{3}} (v_1 - v_0) \right) }\nonumber \\&+ O(\tilde{\chi }^8/\tilde{\mu }_0^4) \end{aligned}$$

where $\tilde{\mu }_0 = \hbox {e}^{-ky_0}$ and where we have taken the limit $\lambda _0, \lambda _1 \rightarrow \infty$ for simplicity. This nuisance is trivially solved by considering the dilaton interactions with gravity of the form

11.7 $$\begin{aligned} \frac{1}{\kappa ^2} \left( \int _{y_0}^{y_1}\,\hbox {d}y\,\hbox {e}^{- 2 A(y)} \right) \sqrt{\hat{g}} \mathcal {R}[\hat{g}]= M_\mathrm{Pl,eff}^2(y_0,y_1) \sqrt{\hat{g}} \mathcal {R}[\hat{g}],\nonumber \\ \end{aligned}$$

which defines the effective Planck scale, as a function of $y_0$ and $y_1$. For a proper holographic interpretation of the effective dilaton potential it is convenient to factor out the dependence of $M_\mathrm{Pl,eff}$ on $y_1$ and reabsorb it on the metric by a transformation $\sqrt{\hat{g}} \rightarrow \sqrt{\hat{g}}/K^2$, which will leave the gravitational kinetic term as $(\mu _0^2/\kappa ^2 k) \sqrt{\hat{g}} \mathcal {R}[\hat{g}]$, with no dilaton contribution (interpreted as a purely elementary operator), and it will bring the dilaton potential to its expected $\chi ^4 F(\chi /\mu _0)$ form, regardless of the identification of $\chi$ and $\mu _0$ as functions of $y_1$ and $y_0$, respectively. We can check this explicitly in the case at hand, where

11.8 $$\begin{aligned}&\frac{1}{k} \int _{y_0}^{y_1}\,\hbox {d}y\,\hbox {e}^{- 2 A(y)}= -\frac{1}{2} (\mu _0^2-\chi ^2)\nonumber \\&\quad + i \frac{\sqrt{2}}{8} \delta ^2 \left[ \mathcal {B}\left( \left( \mu _0^4/\delta ^4 +\sqrt{1+\mu _0^8/\delta ^8} \right) ^2,3/4,1/2 \right) \right. \nonumber \\&\quad - \left. \mathcal {B}\left( \left( \chi ^4/\delta ^4+\sqrt{1+\chi ^8/\delta ^8} \right) ^2,3/4,1/2 \right) \right] \end{aligned}$$

with $\mu _0$ and $\delta$ defined in Eqs. (4.8) and (4.9), respectively, and $\mathcal {B}$ is the incomplete beta function. Evaluating $\delta$ as obtained solving the $\phi$ BCs, Eq. (4.17), one obtains the expected $K= 1 + O(\chi ^2/\mu _0^2)$. Likewise, if the dilaton is parametrized by $\tilde{\chi }$, the effective potential after the rescaling of the metric gets their expected scale invariant form,

11.9 $$\begin{aligned} \frac{\hbox {e}^{-4 A(y_1)}}{K^2} = \tilde{\chi }^4 \left( \mathrm{sech}^2\left( \frac{\kappa }{\sqrt{3}} (v_1 - v_0) \right) +O(\tilde{\chi }^2/ \tilde{\mu }_0^2) \right) ,\nonumber \\ \end{aligned}$$

where the constant factor can be redefined away.

## Appendix F: Linear IR potential

Let us illustrate again our finding with a different IR brane potential, such that its contribution to the effective dilaton potential does not vanish, contrary to the case considered in the main text for $\lambda _1 \rightarrow \infty$. We have

12.1 $$\begin{aligned} V_1(\phi ) = \Lambda _1 + \alpha _1 \phi , \end{aligned}$$

and the same UV brane potential as Eq. (4.2).

The $\phi$ BCs now fix (again in the limit $\lambda _0 \rightarrow \infty$)

12.2 $$\begin{aligned} \phi _0&= v_0 \mu _0^{\epsilon } , \end{aligned}$$

12.3 $$\begin{aligned} \delta&= \chi \left( \frac{4\sqrt{3}k-\sqrt{(4\sqrt{3}k)^2+ (\alpha _1 \kappa )^2}}{\alpha _1 \kappa } \right) ^{1/4} . \end{aligned}$$

The effective potential is

12.4 $$\begin{aligned}&V_\mathrm{UV} = \mu _0^4 \left[ \frac{6k}{\kappa ^2}-\Lambda _0 \right] , \end{aligned}$$

12.5 $$\begin{aligned}&V_\mathrm{IR} = \chi ^4 \left[ a_0 + \alpha _1 v_0 (\chi /\mu _0)^{-\epsilon } \right] , \end{aligned}$$

where

12.6 $$\begin{aligned} a_0&= \Lambda _1 + \frac{\sqrt{3}}{2 \kappa ^2} \sqrt{(4\sqrt{3}k)^2+ (\alpha _1 \kappa )^2}\nonumber \\&+ \frac{\sqrt{3}}{2 \kappa } \alpha _1 \log \left[ \frac{4\sqrt{3}k}{\alpha _1 \kappa +\sqrt{(4\sqrt{3}k)^2+ (\alpha _1 \kappa )^2}} \right] . \end{aligned}$$

It is required that $a_0 > 0$, while $\alpha _1 <0$.

The minimum is found at

12.7 $$\begin{aligned} \frac{\langle {\chi } \rangle }{\mu _0} = \left( - \frac{a_0}{v_0 \alpha _1} \right) ^{-1/\epsilon } + O(\epsilon ) , \end{aligned}$$

while the dilaton mass is

12.8 $$\begin{aligned} m_\chi ^2 = 4 \epsilon a_0 \langle {\chi } \rangle ^2 . \end{aligned}$$

## Footnotes

1 This matching was also recently used by Chacko et al. [18] for finding the solution to a particular bulk potential providing small perturbations around AdS.

2 The UV brane is a source of explicit breaking, which is eliminated once the UV brane is removed, $\mu _0\rightarrow \infty$.

3 The reader may notice that $\hbox {e}^\alpha (a)$ is mildly dependent on $y_1$ so that the scale transformation of the dilaton field is slightly non-linear, $\chi \rightarrow f(\chi )\chi$, with $f(\chi )$ a slowly varying function. One might then argue that a more natural parametrization of the dilaton field is provided by $\chi =\hbox {Exp}[-k y_1]$, which transforms covariantly even though it does not seem to reproduce the expected quartic potential. In fact, in Appendix E, we clarify these points and show how both parametrizations are legitimate and give rise to a purely quartic potential once the kinetic mixing with gravity is properly taken into account.

4 We have dropped a term $\frac{\sqrt{3}}{2 \kappa } \log \left( \tanh \left( 2k(y_\mathrm{c}-y_0)\right) \right)$ which is exponentially small for $y_\mathrm{c} \gg y_0$, but which strictly ensures $\phi (y_0)=v_0$. This term would be automatically included if the matching of the $\phi _\mathrm{r}$ was at $y=y_0$ instead of $y \rightarrow -\infty$. This approximation propagates to Eq. (5.13) and amounts to unimportant $O(\chi /\mu _0)$ corrections.

## References

[1] Fubini S (1976). A new approach to conformal invariant field theories. Nuovo Cim. A.

[2] Holdom B, Terning J (1987). A light dilaton in gauge theories?. Phys. Lett. B.

[3] Holdom B, Terning J (1988). No light dilaton in gauge theories. Phys. Lett. B.

[4] Randall L, Sundrum R (1999). A large mass hierarchy from a small extra dimension. Phys. Rev. Lett..

[5] Goldberger WD, Wise MB (1999). Modulus stabilization with bulk fields. Phys. Rev. Lett..

[6] R. Rattazzi, A. Zaffaroni, Comments on the holographic picture of the Randall-Sundrum model. JHEP **0104**, 021 (2001). hep-th/0012248

[7] N. Arkani-Hamed, M. Porrati, L. Randall, Holography and phenomenology. JHEP **0108**, 017 (2001). hep-th/0012148

[8] Csaki C, Graesser M, Randall L, Terning J (2000). Cosmology of brane models with radion stabilization. Phys. Rev. D.

[9] Goldberger WD, Wise MB (2000). Phenomenology of a stabilized modulus. Phys. Lett. B.

[10] C. Csaki, M.L. Graesser, G.D. Kribs, Radion dynamics and electroweak physics. Phys. Rev. D **63**, 065002 (2001). hep-th/0008151

[11] C. Csaki, J. Hubisz, S.J. Lee, Radion phenomenology in realistic warped space models. Phys. Rev. D **76**, 125015 (2007).0705.3844 [hep-ph]

[12] B. Bellazzini, C. Csaki, J. Hubisz, J. Serra, J. Terning, A higgslike dilaton. Eur. Phys. J. C **73**, 2333 (2013). 1209.3299 [hep-ph]

[13] Z. Chacko, R. K. Mishra, Effective theory of a light dilaton. 1209.3022 [hep-ph]

[14] Z. Chacko, R. Franceschini, R.K. Mishra, Resonance at 125 GeV: Higgs or Dilaton/Radion? JHEP **1304**, 015 (2013). 1209.3259 [hep-ph]

[15] R. Contino, A. Pomarol, R. Rattazzi, talk by R. Rattazzi at Planck 2010, CERN [slides]; talk by A. Pomarol, 2010 Madrid Christmas Workshop [slides]

[16] Weinberg S (1989). The cosmological constant problem. Rev. Mod. Phys..

[17] R. Sundrum, Gravity’s scalar cousin. hep-th/0312212

[18] Z. Chacko, R. K. Mishra, D. Stolarski, Dynamics of a stabilized radion and duality. 1304.1795 [hep-ph]

[19] T. Abe, R. Kitano, Y. Konishi, K.-y. Oda, J. Sato, S. Sugiyama, Minimal dilaton model. Phys. Rev. D **86**, 115016 (2012) 1209.4544 [hep-ph] [Updated results for minimal dilaton model, 1303.0935 [hep-ph]]

[20] W.D. Goldberger, B. Grinstein, W. Skiba, Distinguishing the Higgs boson from the dilaton at the large hadron collider. Phys. Rev. Lett. **100**, 111802 (2008). 0708.1463 [hep-ph] 10.1103/PhysRevLett.100.111802 18517776

[21] J. Fan, W.D. Goldberger, A. Ross, W. Skiba, Standard model couplings and collider signatures of a light scalar. Phys. Rev. D **79**, 035017 (2009). 0803.2040 [hep-ph]

[22] Holdom B (1981). Raising the sideways scale. Phys. Rev. D.

[23] K. Yamawaki, M. Bando, K.-i. Matumoto, Scale invariant technicolor model and a technidilaton. Phys. Rev. Lett. **56**, 1335 (1986) 10.1103/PhysRevLett.56.1335 10032641

[24] Bando M, Matumoto K-I, Yamawaki K (1986). Technidilaton. Phys. Lett. B.

[25] Appelquist TW, Karabali D, Wijewardhana LCR (1986). Chiral hierarchies and the flavor changing neutral current problem in technicolor. Phys. Rev. Lett..

[26] Appelquist T, Wijewardhana LCR (1987). Chiral hierarchies and chiral perturbations in technicolor. Phys. Rev. D.

[27] S.K. Lamoreaux, Phys. Rev. Lett. **78**, 5 (1997) [Erratum-ibid. **81**, 5475 (1998)]

[28] V.M. Mostepanenko, R.S. Decca, E. Fischbach, G.L. Klimchitskaya, D.E. Krause, D. Lopez, J. Phys. A **41**, 164054 (2008). 0802.0866 [hep-th]

[29] N. Kaloper, Phys. Rev. D **60**, 123506 (1999). hep-th/9905210

[30] C. Csaki, J. Erlich, C. Grojean, T.J. Hollowood, General properties of the selftuning domain wall approach to the cosmological constant problem. Nucl. Phys. B **584**, 359 (2000). hep-th/0004133

[31] C. Bender, S. Orszag, *Advanced mathematical methods for scientists and engineers I: asymptotic methods and perturbation theory*. Springer, Berlin (1999)

[32] L. Vecchi, Phys. Rev. D **82**, 076009 (2010). 1002.1721 [hep-ph]

